# Bracing effectiveness in idiopathic early onset scoliosis followed to skeletal maturity: a systematic review and meta-analysis

**DOI:** 10.1007/s43390-025-01043-w

**Published:** 2025-01-22

**Authors:** Matthew Bellamy, Wei Shao Tung, Raveen Jayasuriya, Daniel Hind, Lizzie Swaby, Nikki Totton, Ashley Cole

**Affiliations:** 1 The Medical School, University of Sheffield, Beech Hill Road, Sheffield, S10 2RX UK; 2 Department of Paediatric Orthopaedics and Spinal Surgery, Sheffield Children’s Hospital, Clarkson Street, Broomhall, Sheffield, S10 2TH UK; 3 Sheffield Centre for Health and Related Research, School of Medicine and Population Health, University of Sheffield, Regent Court, 30 Regent Street, Sheffield, S1 4DA UK

**Keywords:** Scoliosis, Spine, Early onset, Brace, Skeletal maturity, Surgery

## Abstract

**Purpose:**

Treating idiopathic Early Onset Scoliosis (idiopathic EOS) is challenging due to ongoing growth and extensive follow-ups. While bracing is effective for Adolescent Idiopathic Scoliosis (AIS), its value for children under 10 remains debated. This systematic review and meta-analysis evaluates the effectiveness of spinal bracing in idiopathic EOS, followed to skeletal maturity.

**Methods:**

We searched Ovid Medline and Web of Science until November 1st, 2023. Studies included idiopathic EOS patients between the ages of 3 and 10 (corresponding to Juvenile Idiopathic Scoliosis), followed to skeletal maturity, with no more than 25% initiating bracing after age 11. The primary outcome was the percentage undergoing scoliosis surgery. Pooled outcomes were calculated using a random effects model and 95% confidence intervals.

**Results:**

Out of 417 studies, 15 met the inclusion criteria, encompassing 868 patients. All were observational with a high risk of bias. The pooled percentage of patients undergoing surgery was 40% (95% CI 27–55%). The percentage of patients with a 5-degree progression or more and those progressing beyond 45 degrees were 44% (95% CI 24–66%) and 33% (95% CI 17–54%), respectively. Factors including larger initial Cobb angles, younger age, smaller in-brace correction, and poor compliance were identified as progression risk factors.

**Conclusions:**

Bracing may prevent progression to surgery in idiopathic EOS when initiated early, but progression and surgery are still more common compared to adolescents. This is the first systematic review and meta-analysis looking at the success of bracing in idiopathic EOS, followed up to skeletal maturity. The high bias and variability of included studies limit the strength of these conclusions, highlighting the need for high-quality research with innovative trial designs.

**Level of evidence:**

IV (systematic review of level IV studies).

**Supplementary Information:**

The online version contains supplementary material available at 10.1007/s43390-025-01043-w.

## Introduction

Idiopathic scoliosis can be divided into subcategories based upon the patient's age at presentation: infantile (0–3 years), juvenile (4–9 years) and adolescent (10–18 years) [1]. More recently, the term Early Onset Scoliosis (EOS) is used to refer to any child under 10 years with scoliosis. Those braced before the age of 4 years, previously called Infantile Idiopathic Scoliosis (IIS) are often treated with plaster jackets (EDF casting) and in 80%, the curve resolves spontaneously without treatment [2]. Idiopathic EOS in patients aged between 4–9 years (previously referred to as Juvenile Idiopathic Scoliosis (JIS)) is more rare than adolescent forms accounting for 13–15% of idiopathic cases [1].

Treatment of idiopathic EOS is usually with a brace to reduce the risk of curve progression and in some cases, improve the curve [3]. If the curve continues to progress, the spinal surgeon needs to decide whether continued bracing will allow curve control until the patient reaches the age of 9 and a definitive posterior instrumented scoliosis correction and fusion can be performed [4]. This decision is largely based on curve flexibility and fixed rotation (rib prominence) rather than the Cobb angle. Growth-friendly surgery maintains some spinal growth, but not to a normal level and has the risks associated with multiple spinal surgeries and at least a 20% chance of unplanned revision surgery due to a problem with implants or infection [5].

Brace treatment has been shown to be effective in randomised controlled trials in Adolescent Idiopathic Scoliosis (AIS), however, no randomised trials have been performed to show the value of bracing in idiopathic EOS [6]. Patients with idiopathic EOS have more growth potential than those with AIS and successful brace treatment is generally considered less likely [7, 8]. It takes many years to successfully brace an idiopathic EOS patient to skeletal maturity, so this length of follow-up is also less commonly reported. Due to the lack of this reliable data, counselling early onset patients on expected progression is often challenging.

In this study, we systematically reviewed the literature and performed a meta-analysis using strict criteria to evaluate the effectiveness of brace treatment started after the age of 3 in idiopathic EOS followed up to skeletal maturity.

## Methods

### Database selection

We performed a systematic review searching Ovid Medline and Web of Science from inception to November 1st, 2023 (Appendix 1, 2). The search strategy for each database was custom created and involved key phrases and words including MESH terms. The search strategy incorporated both “Juvenile Idiopathic Scoliosis” and “Early-Onset Scoliosis” to ensure a comprehensive review of the available literature. The full search strategy can be found in the pre-specified protocol registered on PROSPERO (PROSPERO 2024 CRD42024521818). Reference lists of all included studies were also searched for relevant papers. Trial registries and grey literature were not utilised.

### Inclusion and exclusion criteria

The studies retrieved from the literature search were included in this systematic review according to the following inclusion criteria (Table 1): all patients must have started bracing after the age of 3 and needed to have a diagnosis of idiopathic EOS, or where idiopathic EOS patients were reported separately to the whole cohort (corresponding to JIS). All patients needed to reach skeletal maturity, or those reaching skeletal maturity were reported separately. An existing knowledge of the literature required a plan to deal with studies where some idiopathic EOS patients were braced in adolescence. No more than 25% of patients initiated bracing over the age of 11 or all patients were defined as Risser 0 at the start of bracing treatment if the age at bracing and standard deviation (SD) or range was not given. Bracing age will be calculated from the mean and SD using the normal distribution. Where the SD was not given, this was estimated from the range (min to max) divided by 4. An age of 11 years was chosen as these patients will have the maximal adolescent growth spurt whilst in brace based on previous literature [9]. Patients must be prescribed any form of spinal bracing therapy. Casting treatments were excluded. Patient’s undergoing bracing before the age of 3 were excluded (corresponding to IIS and heterogenous definitions of JIS). Patients with intraspinal pathology, case reports, reviews, protocols, letters, and guidelines were excluded.

**Table 1:** Study inclusion and exclusion criteria

Inclusion criteria	Exclusion criteria
Idiopathic Early Onset Scoliosis (EOS) population	Non-idiopathic EOS aetiology
Bracing initiated after 3 years of age	Over 25% started bracing over the age of 11
Outcomes to skeletal maturity	Skeletally immature at last outcome
No more than 25% initiated bracing over the age of 11	Main structural curves not reported
Rigid or soft brace prescription	Case reports, reviews, or abstracts
	Previous spinal surgery before brace initiation
	Casting or other non-operative therapies
	Idiopathic scoliosis group cannot be separated by aetiology
	Idiopathic EOS braced younger than 3

### Study selection

Two reviewers (MB and WST) blindly examined paper titles and abstracts for their eligibility. After initial screening, full text reads of potential studies were screened for definitive inclusion. Any uncertainty concerning specific studies was reviewed by a third reviewer (AC). Studies where the whole cohort did not meet inclusion, but a smaller cohort could be separately extracted were included.

### Quality assessment and risk of bias

The Methodological Index for Non-Randomized Studies (MINORS) scale was used to assess the methodological qualities of each included study [10]. Following on from our eligibility assessment, two authors (MB and RJ) independently recorded all pertinent data and MINORS score. If there was disagreement, a third author (AC) served as tie breaker. A GRADE-style approach was used by the same reviewers to assess certainty in the body of evidence.

### Data collection

An online collection form was created on Microsoft Excel and was used to record data from the included studies. This included background characteristics (authors, year, publishing journal, curve types, PICO, curve classification), characteristics before bracing (age at diagnosis, age at brace initiation, skeletal age, Cobb angle, intention of brace), characteristics in brace (wear-time prescribed, compliance, correction) and long term follow up data (age, skeletal grade, Cobb angle, surgical correction, resolution or progression defined by the SRS criteria). Data on reported complications and information on the risk of progression and early predictors of success or progression were further isolated using a narrative analysis.

### Outcomes

The primary outcome of our study was the percentage of children with idiopathic EOS braced after the age of 3 who had reached skeletal maturity and received an operation. Secondary outcomes of this study were based around the SRS bracing criteria. Curves that improved greater than 5 degrees, progressed greater than 5 degrees or had no progression within 5 degrees were isolated. Secondary outcomes also included the percentage of patients progressing past 45 degrees to standardise the definition of surgery.

### Meta-analysis

A meta-analysis was used to combine the findings of studies retrieved from the above search. Analysis was completed in R (Version 4.3.2) and used the package “meta”. A single-arm meta-analysis was completed using the data from the fourteen available studies. Heterogeneity was assumed between the studies, which was confirmed during the meta-analyses with high I2 values (91%, 93% and 90% for primary and secondary outcomes respectively), therefore a random-effects model was used. As a sensitivity analysis, the impact of potentially important covariates was assessed within a mixed-effect regression model. The weighted random effects model was fit, followed by a covariate adjusted version of the model. The covariates included were mean age at the start of bracing and the mean Cobb angle at brace initiation. The resulting models were assessed using the Akaike Information Criterion (AIC) to assess the fit of the data.

## Results

**Fig. 1:**
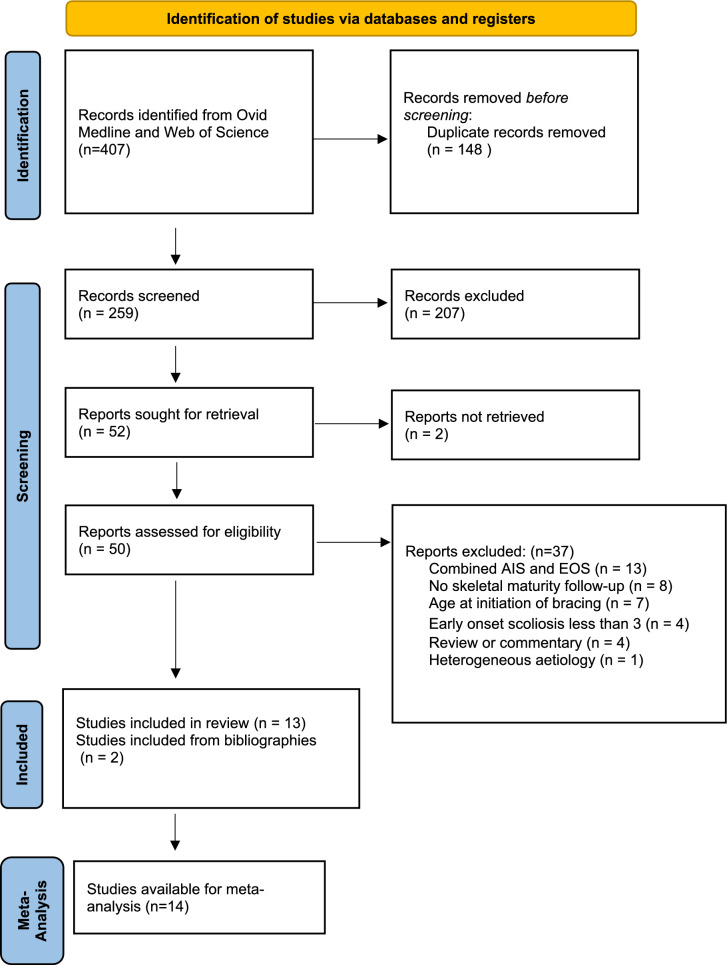
PRISMA 2020 flow diagram for new systematic reviews which include searches of databases and registers

Our initial database search revealed 407 potentially relevant papers (Fig. 1). After duplication removal, 259 papers were screened by their title and abstracts. After full text review, 37 papers were excluded leaving 13 papers [3, 4, 11–21]. Two further papers were added from the bibliography screening of all included articles (Table 2) [22, 23]. Additionally, 2 of the papers were written by the same author and we believe studied the same population of patients with different outcomes [3, 15]. Due to our inclusion criteria being met by both studies and thus having relevant data to skeletal maturity, both studies are included in the systematic review but only one study by that author was included in the meta-analysis. Furthermore, the data from the 14 eligible studies (*n* = 741) included 13 studies with relevant primary outcome data for meta-analysis and secondary outcome data from 9 studies.

**Table 2:** Characteristics of included studies

Authors	Year	Type of Brace	Number of Patients	Mean Age at Brace Initiation	% Braced Under 10	Mean Cobb Angle Before Bracing	Indication for Bracing
Tsirikos	2023	Boston	45	7.8	100	NR	Cobb angle: 20–40 degrees
Babaee	2020	Milwaukee (88%) TLSO (12%)	75	8.6	70.5	34.1	Cobb angle: > 20 degrees
Harshavardhana	2017	Custom TLSO or Milwaukee	93	NR*	100	NR	Cobb angle: > 20 degrees
Fusco	2014	Spinecor, Sibilla, Lyon and Sforzesco	30	NR*	NR*	23.2	Risser 0, Cobb angle: 20–30 degrees
Aulisa	2014	Progressive Acting Short Brace (PASB), Milwaukee, Lyon	113	8.1	100	29.6	Cobb angle: 20–40 degrees (progression > 5 degrees between 20–25 degrees)
Aulisa	2014	Progressive Acting Short Brace (PASB), Milwaukee, Lyon	127	9	79.8	29.5	Cobb angle: 20–40 degrees (progression > 5 degrees between 20–25 degrees)
Coillard	2014	SpineCor	63	NR*	100	28.1	Cobb angle > 15 degrees with progression
Khoshbin	2014	TLSO, Milwaukee, and Charleston	88	9.3	68	31	Cobb angle: > 20 degrees
Jarvis	2008	Charleston	23	10.3	50	30	Cobb angle: > 20 degrees
Mannherz	1996	Localiser jacket or Milwaukee	31	9	72	NR	Cobb angle: < 40 degrees
Whitaker	2022	Boston (90%) and Charleston (10%)	91	7.9	100	30	Cobb angle: > 20 degrees
Verhofste	2020	Boston (94%), Rigo–Cheneau brace (6%)	20	7.9	100	43.9	Cobb angle: > 40 degrees
Kahanovitz	1982	Milwaukee	15	7.5	100	37.2	Cobb angle: > 20 degrees
Emans	1985	Boston	34	NR*	100	NR	Cobb angle: > 20 degrees and progression > 10 degrees
Keiser	1975	Milwaukee	20	NR*	NR*	38	Cobb > 20 degrees

^*^Not reported (risser 0 at initiation)

All 15 studies were prospective or retrospective case series. JIS was the focus in 11 studies, mixed idiopathic scoliosis in 3 studies, and a heterogeneous group of scoliosis in 1 study. The sample size from included studies ranged from 15 to 127 patients with 868 included patients in the review. Mean age at diagnosis was 7.7 years with a mean age at brace initiation of 8.5 years. Rigid full-time bracing was reported by 13 studies, 1 reported on rigid night-time bracing, 1 used soft full-time braces and 1 used a mix of rigid and soft braces. The most common indication for bracing initiation was a Cobb angle above 20 and below 45 degrees.

Only 3 studies reported on complete SRS bracing guidelines. Stabilisation or progression but no improvement was reported by 3 further studies and 3 more studies reported on progression alone. The remaining studies did not report any data on improvement, correction, or stabilisation. No study objectively measured wear time or compliance. Summary characteristics of the studies are given in Table 2.

Concurrent spinal pathology and underlying conditions were reported by 6 studies. Only 1 study provided a detailed breakdown of comorbidities, with the most common including leg length discrepancy, developmental dysplasia of the hip and unspecified neurological conditions. The remaining 5 studies acknowledged the importance of comorbidities but did not specify any underlying diagnoses. All 6 studies excluded patients with intraspinal pathology requiring intervention.

Of the 15 studies, only 3 mentioned MRI assessment, all of which used MRI to exclude intraspinal pathology requiring intervention. Two studies reported no intraspinal pathology in their cohorts. One study found that 23% of idiopathic EOS patients had MRI-detected pathology, including syrinx (unrelated to Chiari malformation), fatty filum, prominent central canal, and low-lying conus. In this study, 13% of patients had intraspinal pathology that did not require surgical intervention, so these patients were braced and treated similarly to those with idiopathic EOS.

### Quality assessment

**Fig. 2:**
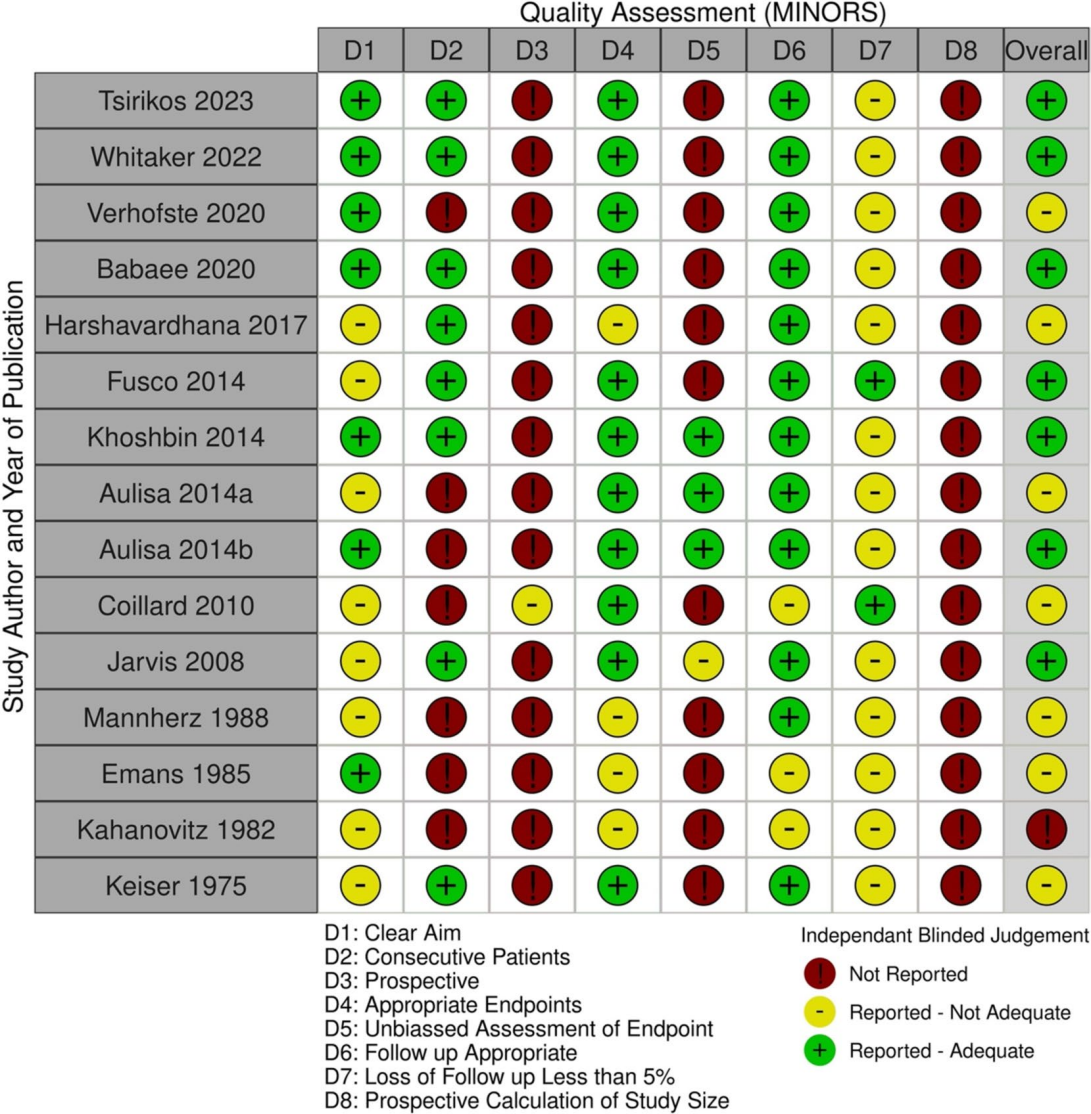
A representation of the quality assessment for all included studies

Quality assessment using the MINORs criteria demonstrated that all studies had low to moderate scores indicating poor methodological study design (Fig. 2). Mean scores were 8 with a range of 4–11. A handful of studies claimed to be prospective in nature but still utilised retrospective databases. Furthermore, only 3 studies adequately reported unbiased end point assessments. There was no sample size calculation in any study. All studies had a GRADE rating of “very low”. This is due to all studies being observational in nature with no control group. All studies demonstrated a high risk of selection bias, performance bias, and publication bias. Furthermore, all studies demonstrated small sample sizes and lack of precision in estimating treatment outcomes for a generalisable population. Therefore, these findings should be interpreted with clinical caution and may be subject to significant uncertainty.

### Primary outcome: patients requiring surgery after bracing

There were 13 studies with follow up to skeletal maturity that met the inclusion criteria, which yielded 741 patients. The indication for surgery varied significantly between studies. The indication for surgery was greater than 45 degrees in 7 studies, greater than 50 degrees in 4 studies and over 60 degrees in 2. Two further studies did not report an indication and the final study reported an indication of 60 degrees for thoracic curves and 45 degrees for thoracolumbar or lumbar curves.

**Fig. 3:**
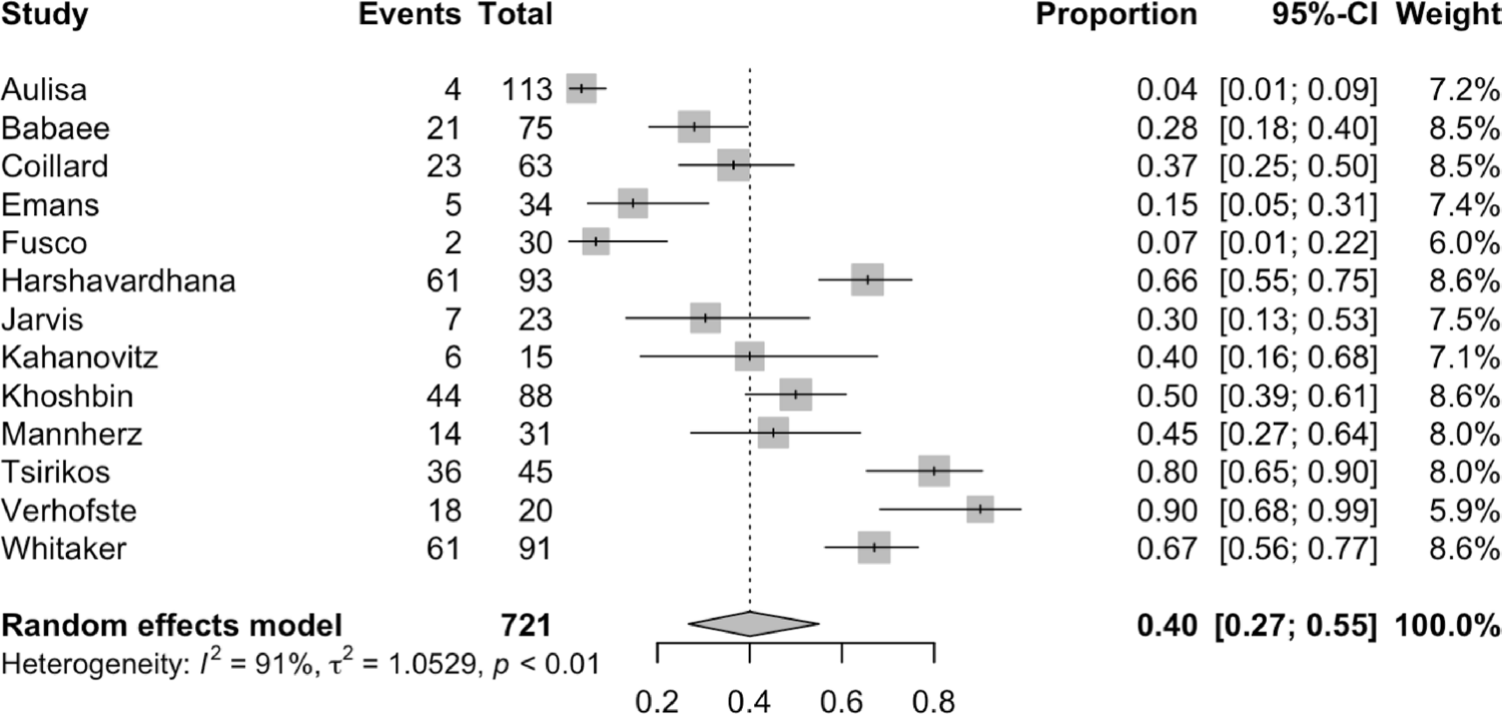
A forest plot of relevant studies demonstrating patients requiring scoliosis surgery after bracing

The percentage of patients needing surgery following bracing ranged from 4 to 90% in the included studies. The random effects meta-analysis model included 302 instances of patients needing surgery, resulting in an overall percentage of 40% (95% CI 27–55%). The forest plot can be found in Fig. 3. The covariates were not found to be important within the regression model, therefore covariate adjustment was not required.

## Secondary outcomes

### Curve progression by greater than 5 degrees

**Fig. 4:**
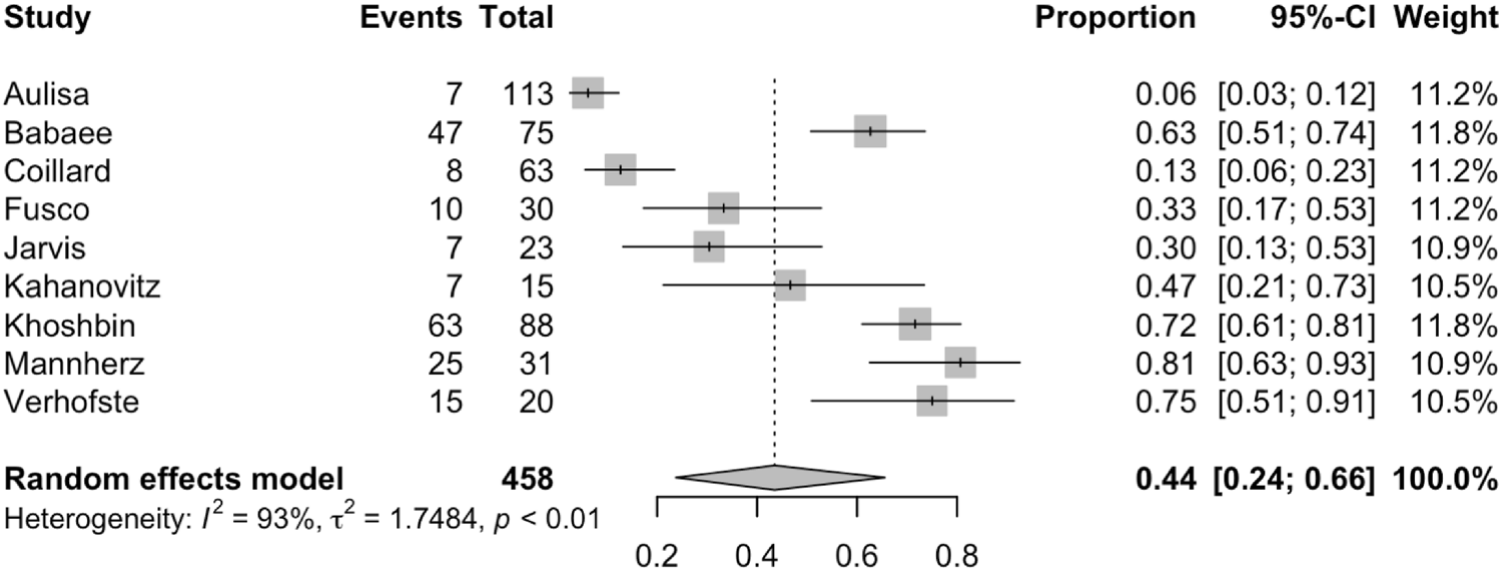
A forest plot of relevant papers demonstrating curve progression by greater than 5 degrees

There were 9 studies comprising 458 patients which reported on curve progression of 5 degrees or more. The percentage of patients that progressed by 5 degrees or more following bracing ranged from 6 to 81%. The overall percentage from the random-effects model is 44% (95% CI: 24% to 66%) which consisted of 189 instances. The forest plot is shown in Fig. 4. As with the primary analysis, the addition of the covariates of interest was not found to be important.

### Curve progression above 45 degrees

**Fig. 5:**
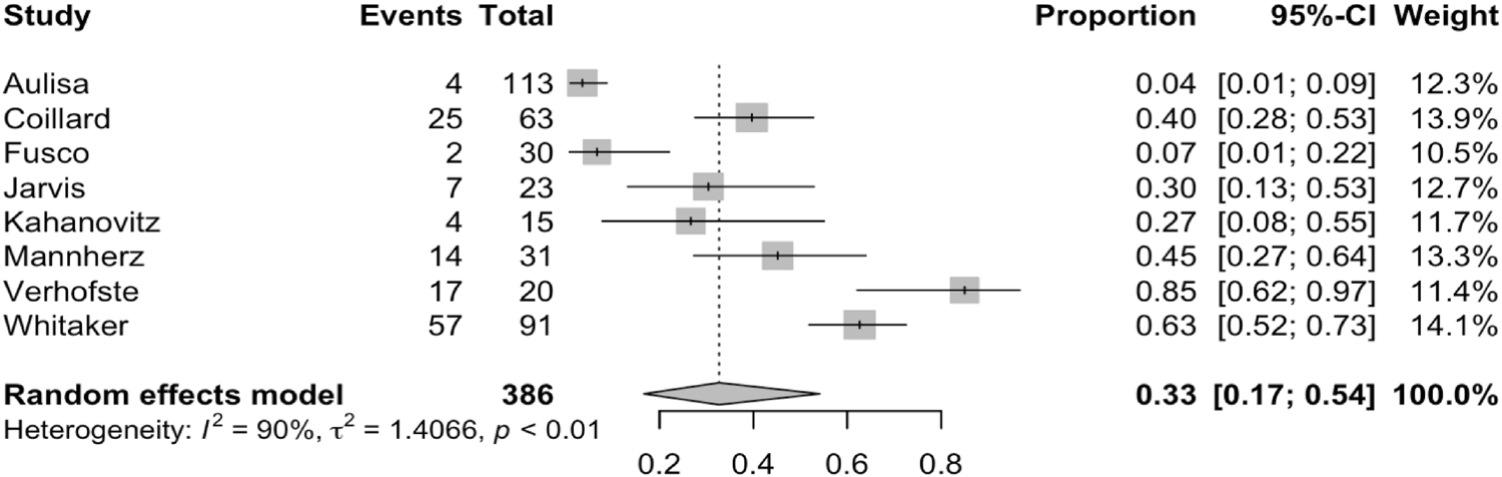
A forest plot of relevant papers demonstrating curve progression above 45 degrees

There were 8 studies comprising 386 patients reporting on Cobb angle progression above 45 degrees. This magnitude was chosen as it was the most reported definition of timing for surgical intervention. The percentage of patients that progressed up to 45 degrees following bracing ranged from 4 to 85% from the 8 studies. The overall percentage from the random-effects model is 33% (95% CI 17–54%) which consisted of 130 instances. The forest plot is shown in Fig. 5. Sensitivity analyses had indicated once again, no change to the model with the inclusion of covariates of interest.

### Early predictors of success or progression

The addition of covariates including age at brace initiation and Cobb angle at brace initiation were not found to be important within the regression model of the available data. Characteristics known to be important prognostic factors in brace treatment of AIS such as compliance, brace design, in-brace correction and SRS reporting criteria outcomes were collected [24]. However, due to the heterogeneity of the studies and the lack of standardised reporting, these factors could not be included in the meta-analysis.

A qualitative review of the literature found an increased risk of progressive curves in patients with: larger presenting Cobb angles before brace initiation (10 papers); less brace compliance (6 papers); younger age at bracing (5 papers); night-time bracing or non-custom fabricated rigid braces (5 papers); and low in-brace correction (3 papers). Furthermore 7 papers commented on curve type, demonstrating that double major curves and large single thoracic curves often had the least in-brace correction and often progressed to surgery.

### Complications from bracing

Only two studies reported adverse outcomes from bracing. A consistent finding of vertebral body half wedging was noted on the side of the concavity in one paper. The other paper stated that orthodontic complications, pressure sores and brace breakage were all complications from Milwaukee brace treatment in this population. No studies mentioned any respiratory complications or respiratory decline during or after bracing treatment.

## Discussion

With a large range of reported success from different studies, counselling patients on bracing before their growth spurt can be challenging. We chose brace treatment staring before the age of 11 in at least 75% of the patients as our inclusion criteria cut-off as this was considered before the age of onset of the adolescent growth spurt in the majority of girls and all boys [9]. There is also evidence that idiopathic EOS patients braced after aged 10 have the same degree of curve progression as brace treated AIS patients although more will progress to greater than 45 degrees [25]. Nearly 90% of surgically treated curves show significant progression during this key phase of maximal growth velocity [7, 9].

The primary outcome of our study was the percentage of patients requiring surgery after bracing. Surgical intervention in the studies included in our review of 13 papers ranged from 4 to 90% demonstrating a large variation in outcomes. Our analysis shows that on average, 40% of brace treated idiopathic EOS patients will have surgery (95% CI 27–55%). In comparison, the BRAIST randomised control trial demonstrated that bracing AIS resulted in 28% of patients progressing past the surgical threshold of 50 degrees [6]. The meta-analysis by Zhang and Li [28] found 32% of brace treated AIS patients progressed to surgery [26]. It is therefore likely that brace treatment in the early onset cohort is less likely to avoid surgical treatment than in AIS, but success rates are reasonable at 60% (95% CI 45–73%).

Secondary outcomes of interest were progression greater than 5 degrees and curves progressing over 45 degrees, which is a commonly considered surgical threshold. Our analysis showed that progression greater than 5 degrees ranged from 6 to 81% with a pooled estimate of 44% for early onset patients (95% CI 24–66%). As expected, more patients showed curve progression by more than 5 degrees than progressed to surgery. However, if the 9 papers available for the pooled analysis of curve progression by more than 5 degrees had been used in the analysis of curve progression to surgery, 30% would have progressed to surgery compared to the 44% progressing by more than 5 degrees. This shows a wider gap, as would be expected, between curves progressing by more than 5 degrees and curves progressing to surgery. A recent systematic review of different brace types found progression greater than 5 degrees in 27% of AIS patients treated with a rigid full-time brace, again suggesting that curve progression during bracing for idiopathic EOS is likely to be more common than in AIS [27].

Curve progression to more than 45 degrees ranged from 4 to 85% in the 8 studies with a pooled percentage of 33% of braced idiopathic EOS patients. If these 8 studies were used in the primary analysis of progression to surgery, 35% would have progressed to surgery in the pooled analysis. This demonstrates how the chosen studies can significantly influence the success rates for bracing in idiopathic EOS due to the wide range of values between papers.

Unfortunately, many of the planned clinical parameters, including the SRS bracing criteria, could not be used in the meta-analysis due to a lack of standardised reporting or un-reported data. We did not include brace design in our analysis due to many studies using a mixture of European and Boston bracing in the same cohort of patients. However, isolating predictive factors of bracing success is important for the counselling and monitoring of curve progression [24]. In our review there is a suggestion that larger presenting Cobb angles, poor compliance, younger age at brace initiation, brace design (night-time and non-custom) and poor in-brace correction led to greater progression. These factors are similar to those found in adolescent populations where in-brace correction and brace compliance are probably the strongest predictors of bracing success [28]. However, additional factors can make compliance and brace design difficult in this younger cohort. Early onset patients are likely to spend more years in brace, going through all the challenges of growing up and schooling wearing a brace. Lin et al. (2019) demonstrated that bracing interventions and longer time in-brace have been correlated to an increase in depressive symptoms in juveniles and adolescents [29]. For younger patients, parent support also plays a vital role in encouraging compliance and supporting positive attitudes [30].

Accurately measuring the quantity of brace-wearing using temperature sensors is becoming more common in studies, with increased attention to also measure the quality of brace wearing using pressure sensors [31]. None of the studies in this review reported on the quantity or quality of brace wear. Future studies should prioritise measuring brace compliance and possibly the quality of brace wearing.

The complication data in our review was notably limited. No studies reported on respiratory function in braced idiopathic EOS patients, despite this being a significant side effect and drawback of brace effectiveness in this young population. Furthermore, the only complication data available related to the Milwaukee brace, which is rarely used in routine clinical practice today. Future studies should prioritise comprehensive reporting of complications from both clinicians and patients, as well as including pulmonary function assessments in this developing age group.

To our knowledge, this is the first systematic review and meta-analysis that collates the current literature surrounding the outcomes and effectiveness of bracing therapy in a true idiopathic EOS population followed up to skeletal maturity. Bracing treatment for early onset patients is often undertaken for many years longer than their adolescent peers, with an impact on function and mental health, so determining the long-term effectiveness remains of high importance [32]. Up to this point, only small, retrospective studies with a high risk of bias have been published. A randomised controlled trial of bracing in idiopathic EOS is unlikely, due to the long follow-up required and the difficulties in surgeon and patient equipoise on treatment options. We must also recognise that brace treatment in this population can be divided into 2 groups: (1) those braced to try to correct the curve or reduce the risk of progression to surgery in curves 20–40 degrees [6]; and (2) those braced with a lower chance of avoiding surgery with the main aim to delay surgery, ideally to allow primary instrumented fusion and avoid growth-friendly instrumentation. Future studies should report separately for the patients in each of these 2 groups and only include patients braced before the age of 10 years.

Furthermore, many of the often-cited articles with title focussed on bracing idiopathic EOS patients or JIS are in fact initiating the bracing treatment in the adolescent years (> 10), which falls after the peak pubertal growth spurt (Table 4) [9]. Future studies should report curve change to skeletal maturity and up to 2 years after skeletal maturity for curve progression > 5 degrees, no change, improvement > 5 degrees; progression to 45 degrees or more; progression to 50 degrees or more; and surgery required before skeletal maturity. Surgery should be reported as definitive fusion or growth-friendly surgery with careful documentation of the outcome of surgery and any further planned and unplanned surgical procedures. Studies should also collect patient questionnaires on quality of life (pain, function and mental health), pulmonary function and document any adverse events and loss to follow-up. Possible confounding variables should be collected, including Cobb angle and age at brace initiation, age at diagnosis, brace design and in-brace correction. There should also be an objective measure of brace compliance such as temperature sensors and possibly tension meters. These recommendations follow the SOSORT recommendations for research [33], SRS bracing criteria [34] and best practice guidelines for bracing [35].

### Limitations

Firstly, the included studies are of low quality with a high risk of bias. There is a large heterogeneity amongst studies with marked differences in bracing protocols, end points and outcomes (Table 3). Due to our strict inclusion criteria, some larger and frequently cited trials were not included due to their age at the initiation of bracing being outside of the early onset years (Table 4). Furthermore, every effort was made to ensure all potential papers were included but due to the nature of systematic reviews it is possible that our search strategy may not have identified all eligible studies. Additionally, many of the papers did not focus on the early onset population in isolation and so separate calculations were deduced from the published results. The heterogenous mixture of brace designs and no unbiased compliance reporting, or the average wear time may influence the effectiveness of the braces prescribed. Many of these factors have been found to be significant predictors of bracing outcomes in previous literature despite not being significant covariates in our model, indicating that more robust data is needed to make meaningful conclusions. Finally, we selected progression to a surgical threshold as our primary outcome due to the absence of curve magnitude data at key time points in some studies, such as at the initiation of bracing and at skeletal maturity. This lack of data makes it difficult to quantitatively assess curve correction in accordance with SRS and SOSORT reporting guidelines [34, 36].

**Table 3:** Included study limitations and loss to follow up

Authors	Year	% Lost to follow up	SRS criteria followed	Study limitations
Tsirikos	2023	48%	No	Separate reporting of lumbar and thoracic curves. Curves over 50 degrees braced so progression over this threshold is difficult to determine
Babaee	2020	54%	No	Not all patients had bracing initiated in the early onset years
Harshavardhana	2017	27%	No	Not all patients had bracing initiated in the early onset years. Limited reports of patients exclusively braced under 10
Fusco	2014	NR	No	Bracing treatment combined with exercise programme. Bracing assumed to start at diagnosis of scoliosis
Aulisa	2014	24%	Yes	No in-brace measurements reported although reported to have measured
Aulisa	2014	NR	No	Combines EOS and AIS for key outcomes
Coillard	2014	16%	No	Definition of surgical threshold is different for thoracic and lumbar curves
Khoshbin	2014	NR	No	No Cobb angle measurements at skeletal maturity reported
Jarvis	2008	6%	No	Bracing assumed to start at diagnosis of scoliosis
Mannherz	1996	3%	No	Observational study design where different groups received differing treatment programmes
Whitaker	2022	14%	Yes	Mixture of night and day braces. Final reporting missing 8 patients
Verhofste	2020	NR	Yes	Bracing assumed to start at diagnosis of scoliosis
Kahanovitz	1982	NR	Yes	Cobb angle at skeletal maturity includes those that underwent spinal surgery
Emans	1985	NR	No	Majority of the paper describes AIS and EOS mixed
Keiser	1975	< 19%	No	Definition of skeletal maturity is very vague. Bracing assumed to start at diagnosis of scoliosis

**Table 4:** Excluded but often referenced studies with reason for exclusion

Authors	Year	Cohort	Exclusion reason
Sauvagnac	2022	45	No distinction between EOS and AIS
Heemskerk	2020	49	52% of patients above 11 when braced
Lin	2017	96	Not all patients reached skeletal maturity when outcomes calculated
Sewell	2017	30	Not all patients reached skeletal maturity when outcomes calculated
Van Hessem	2014	4	Not all patients reached skeletal maturity when outcomes calculated
Masso	2010	34	33% of patients above 11 when braced
Robinson	2002	109	Not all patients reached skeletal maturity when outcomes calculated
Tolo	1982	42	Not all patients reached skeletal maturity when outcomes calculated
Figuerido	1981	45	Not all patients reached skeletal maturity when outcomes calculated

## Conclusions

This systematic review and meta-analysis of 13 included studies demonstrates low quality evidence supporting the use of bracing in idiopathic EOS with 40% of patients having surgical treatment before skeletal maturity (95% CI 27–55%). Brace success is likely to be lower than that observed in AIS. These results can be used to aid shared decision making and advise EOS patients and families on the likelihood of progression and surgical intervention when bracing is initiated before puberty (noting the relatively wide confidence limits). There is a need for further high-quality research in this area, evaluating curve progression, quality of life, and the effects of possible confounding variables that may influence the success and failure of bracing. The findings presented aim to demonstrate the need for further high-quality research, guide sample size calculations, inclusion and exclusion criteria, outcome measures and the methodological design of future clinical trials to address these gaps.

## Supplementary Information

Below is the link to the electronic supplementary material.

Supplementary file1 (DOCX 16 KB)

Supplementary file2 (DOCX 15 KB)

## Data Availability

All data utilized in this review and meta-anlysis is publicly available.
