# Supplementary material for: Bracing effectiveness in idiopathic early onset scoliosis followed to skeletal maturity: a systematic review and meta-analysis
Source: Spine Deform. 2025 Jan 22;13(3):939–50. doi: 10.1007/s43390-025-01043-w (PMC12021973; doi:10.1007/s43390-025-01043-w)
Supplement: Supplementary file 1 — Supplementary file1 (DOCX 16 KB) [file 43390_2025_1043_MOESM1_ESM.docx]

| Medline (via <https://pubmed.ncbi.nlm.nih.gov/>) accessed on 01/11/2023 | | |
| --- | --- | --- |
|  | Search Terms | Results |
|  | scoliosis.mp. or Scoliosis/ | 29938 |
|  | spinal curvature.mp. | 951 |
|  | juvenile.mp. | 97489 |
|  | juvenile idiopathic scoliosis.mp. | 90 |
|  | jis.mp. | 530 |
|  | early onset.mp. | 47852 |
|  | EOS.mp. | 6716 |
|  | orthotic device.mp. or Orthotic Devices/ | 7075 |
|  | brace".mp. or Braces/ | 12059 |
|  | bracing.mp. | 4312 |
|  | 1 or 2 | 30464 |
|  | 3 or 4 or 5 | 97989 |
|  | 6 or 7 | 53404 |
|  | 8 or 9 or 10 | 20568 |
|  | 12 or 13 | 150264 |
|  | 11 and 14 and 15 | 218 |

Appendix 1 Custom Created Medline Search Strategy
