# Supplementary material for: Bracing effectiveness in idiopathic early onset scoliosis followed to skeletal maturity: a systematic review and meta-analysis
Source: Spine Deform. 2025 Jan 22;13(3):939–50. doi: 10.1007/s43390-025-01043-w (PMC12021973; doi:10.1007/s43390-025-01043-w)
Supplement: Supplementary file 2 — Supplementary file2 (DOCX 15 KB) [file 43390_2025_1043_MOESM2_ESM.docx]

| Web of Science (via https://mjl.clarivate.com/search-results) accessed on 01/11/2023 | | |
| --- | --- | --- |
|  | Search Terms | Results |
|  | (TS=(Scoliosis) OR TS=(Spinal Curvature)) | 48796 |
|  | ((((TS=("Juvenile idiopathic scoliosis")) OR TS=("JIS")) AND TS=("juvenile")) | 149 |
|  | ((TS=("EOS")) OR TS=("Early Onset Scoliosis")) | 35957 |
|  | ((TS=(Orthotic Devic*)) OR TS=(Brac*)) OR TS=(Bracing) | 2863538 |
|  | #2 OR # 3 and Preprint Citation Index (Exclude - Database) | 34237 |
|  | (#1 AND #5 AND #4) | 189 |
|  | #6 | 189 |

Appendix 2 Custom Created Web of Science Search Strategy
